# High-Resolution Harmonics Ultrasound Imaging for Non-Invasive Characterization of Wound Healing in a Pre-Clinical Swine Model

**DOI:** 10.1371/journal.pone.0122327

**Published:** 2015-03-23

**Authors:** Surya C. Gnyawali, Kasturi G. Barki, Shomita S. Mathew-Steiner, Sriteja Dixith, Daniel Vanzant, Jayne Kim, Jennifer L. Dickerson, Soma Datta, Heather Powell, Sashwati Roy, Valerie Bergdall, Chandan K. Sen

**Affiliations:** 1 Comprehensive Wound Center, Davis Heart and Lung Research Institute, Centers for Regenerative Medicine and Cell Based Therapies, The Ohio State University, Columbus, Ohio, United States of America; 2 Veterinary Preventive Medicine, The Ohio State University, Columbus, Ohio, United States of America; 3 Department of Biomedical Engineering, The Ohio State University, Columbus, Ohio, United States of America; 4 Department of Materials Science and Engineering, The Ohio State University, Columbus, Ohio, United States of America; Medical College of Georgia, UNITED STATES

## Abstract

This work represents the first study employing non-invasive high-resolution harmonic ultrasound imaging to longitudinally characterize skin wound healing. Burn wounds (day 0-42), on the dorsum of a domestic Yorkshire white pig were studied non-invasively using tandem digital planimetry, laser speckle imaging and dual mode (B and Doppler) ultrasound imaging. Wound depth, as measured by B-mode imaging, progressively increased until day 21 and decreased thereafter. Initially, blood flow at the wound edge increased up to day 14 and subsequently regressed to baseline levels by day 21, when the wound was more than 90% closed. Coinciding with regression of blood flow at the wound edge, there was an increase in blood flow in the wound bed. This was observed to regress by day 42. Such changes in wound angiogenesis were corroborated histologically. Gated Doppler imaging quantitated the pulse pressure of the primary feeder artery supplying the wound site. This pulse pressure markedly increased with a bimodal pattern following wounding connecting it to the induction of wound angiogenesis. Finally, ultrasound elastography measured tissue stiffness and visualized growth of new tissue over time. These studies have elegantly captured the physiological sequence of events during the process of wound healing, much of which is anticipated based on certain dynamics in play, to provide the framework for future studies on molecular mechanisms driving these processes. We conclude that the tandem use of non-invasive imaging technologies has the power to provide unprecedented insight into the dynamics of the healing skin tissue.

## Introduction

Chronic wounds are major burdens on patients and health care support systems. Every year in the United States, conservative estimates put the number of cases of chronic wounds at more than 6.5 million with a cost burden of over 50 billion dollars [1,2]. Measurement of wound depth, angiogenesis and scar formation are important for the proper assessment and management of the healing wound in the patient [3]. Currently, these measurements require repeated biopsies that necessitate the removal of a portion of the wound to assess biomechanics, morphology and biochemical properties. The invasiveness of this current standard in clinical assessment of wounds perturbs the wound healing process and is also an added burden on the patient. Tissue characterization parameters involving non-invasive methods have been applied to pathological studies of various organs such as the breast, heart and liver [3–6]. However, the diagnostic applications of non-invasive methods involving ultrasonic measurements have not been widely applied to studies involving the skin, particularly in the context of wound healing [7].

In this study we have assessed the applications of a combination of advanced ultrasound based measurements along with laser speckle perfusion imaging to capture the sequence of events as related to the physiological processes of healing in an acute burn wound. We expect that the same measurements could also be applied to capture differences in the physiology of chronic wounds. Laser speckle perfusion imaging (LSI) is a technique that visualizes tissue blood perfusion in the microcirculation in real time. The LSI system provides dynamic response and spatial resolution in the instrument, providing both real-time graphs and video recordings of the area of interest. Dedicated application software enhances the collection and post-processing of images. The speckled patterns (dark and bright areas) generated reflect the degree of movement in any particular area [8,9]. Speckle patterns blur in the region where particles in the blood are in motion. Blurred areas in motion give rise to contrast over the areas outside blood vessels without motion. The blurred micro vessels are color-coded to generate perfusion maps. It is therefore a powerful approach for blood perfusion imaging.

Ultrasonic techniques have been used to quantify physical parameters of biological tissue through measurements of acoustic propagation properties such as velocity, attenuation, absorption and scattering [5]. The basic principle of ultrasound imaging is the use of high frequency sound waves to generate images of internal organs and tissues via a pulse-echo sequence. Modern ultrasound systems have numerous and diverse applications including vascular imaging, visualizing 3D structures in motion and measuring the stiffness of tissues. The ultrasound transducer generates pulses that pass through tissue and reflect back producing echoes. The echoes of reflected and scattered ultrasound waves from tissue boundaries and within tissues respectively result in a B-mode image. The amplitude of the echo relates to brightness of the image [10]. Diagnostic ultrasound techniques typically have noise artifacts and clutter representing undesirable echoes from tissue interfaces. However, ultrasonic imaging of tissue using harmonics has been shown to reduce clutter and markedly improve image quality. Confining the imaging to the harmonic range eliminates much of the near-field artifacts associated with typical ultrasound imaging.

Elastography, also known as elasticity imaging, stiffness imaging or strain imaging, is a dynamic technique that uses ultrasound to non-invasively assess the mechanical stiffness of tissue by measuring tissue distortion in response to external stretch [11,12]. The transducer is used to apply mechanical stress on the tissue by alternative compression and decompression of the skin, and this stress, measured as axial displacement of tissue, is displayed as an elastogram. The elastogram is represented as a color map with a range of colors from red (soft/high strain) to green (intermediate/equal strain) to blue (hard/no strain). This data can also be semi-quantitated using a visual scoring system based on the colors or using strain-ratio measurements usually provided in the elastography software [13]. Color Doppler based detection and analysis of blood flow velocity for high resolution imaging of tissues such as the skin is another unique feature. There are many advantages to using harmonic ultrasonic techniques for analysis of the skin in contrast to deeper organs. Due to the low depth of penetration required, lower frequencies can be used, permitting higher spatial resolution of the sample being analyzed. In skin, higher spatial resolution allows the differentiation of the epidermis, dermis and subcutaneous fat and the muscle layer. This technique has been demonstrated to be a rapid, accurate and non-invasive diagnostic tool in animal models [14].

In the current study, we explored the application of a combination of the ultrasound imaging system with laser speckle perfusion measurements to non-invasively monitor the process of wound healing, including measurements of tissue elasticity and microcirculation. Our intent was to validate such findings against invasive histological and biomechanical data and therefore we adopted a pre-clinical swine model which is known to be powerful in representing the human cutaneous wound [14–17].

## Materials and Methods

### Swine Skin Full Thickness Burn Model

Animal wounding and maintenance procedures were performed as reported previously [17]. Briefly, six domestic Yorkshire white swine (70–80 lbs.) were used in this study for measurements using a set of six burn wounds each. The pig was sedated by Telazol (Fort Dodge Animal Health, Fort Dodge, IA) and anesthetized using isoflurane (3–4%). The dorsal region was shaved and skin was surgically prepared with alternating 2% chlorhexidine and 70% alcohol (Butler Schein, Columbus, OH) scrubs. Under such aseptic conditions, six 1”×1” burn wounds (three on each side of the dorsal spinal area) were made using a burn wound device heated up to 150°C and applied for 60 seconds under standardized pressure conditions [17]. Wounds were dressed with Tegaderm (3M, St. Paul, MN) after burning. Tegaderm dressings were held in place using V.A.C. drape (Owens & Minor, Mechanicsville, VA) and then wrapped with Vetrap and Elastikon (3M, St. Paul, MN). The animal received a single intramuscular injection of buprenorphine (0.3 mg/ml) during recovery from anesthesia and a transdermal fentanyl patch (100 μg/h) was applied to the inner surface of the pinnae. The pig was fed Mazuri laboratory swine chow (non-antibiotic) *ad libitum*, fasted overnight before the procedures, and housed individually in our animal facilities (University Laboratory Animal Resources, Ohio State University). The pig was maintained on 12h light—dark cycles and was euthanized after the completion of experiments on day 42. Laser speckle perfusion images, digital images, and ultrasound images were acquired from the pig pre-burn, immediately post-burn, and on days 3, 7, 14, 21, 28, 35, and 42 post-burn. Wound tissue from the left side of the mid-dorsum was collected on day 14 post-burn and wounds from the right side were collected on day 42 for immuno-histochemical assessments. In addition, tissue was collected for tensile strength measurements on day 42 post burn. Following the completion of the experiments, the animals were euthanized using concentrated KCl (1–2 mmol/kg) (as per IACUC policy:http://orrp.osu.edu/files/2013/07/045-01-Use-of-Pharmaceutical-and-Non-Pharmaceutical-Compounds.pdf) given intravenously (IV) while the animal was held under general anesthesia. This method of euthanasia is in compliance with the AVMA Guidelines and in accordance with OSU-IACUC policy on Euthanasia.

### Ethics statement

All experiments were approved by The Ohio State University Institutional Laboratory Animal Care and Use Committee (Protocol Number: 2008A0012-R2). This study was carried out in strict accordance with the recommendations in the Guide for the Care and Use of Laboratory Animals of the National Institutes of Health. All procedures were performed under 3–4% isoflurane anesthesia and all efforts were made to minimize suffering.

### Biopsies

On the designated days (days 14 and 42 post-burn), swine were anesthetized as described in the burn method, the bandages were gently removed, followed by collection of full thickness wound-edge tissue biopsies using an 8 mm sterile biopsy punch for tissue analyses [17]. Two full thickness excisional biopsies across the whole burn wound length and comparable normal skin strips were collected on days 3, 7 (immuno-histochemistry only), 14 and 42 post-burn for histology, immuno-histochemistry, elasticity and tensile strength measurements. Buprinophine analgesia was provided at the time of biopsy collection, and wound sites re-bandaged as described earlier.

### Histology

Formalin-fixed, paraffin-embedded or optimum cutting temperature (OCT)-embedded frozen wound-edge specimens were sectioned (10 μm) [14–17]. The paraffin sections were deparaffinized and stained with hematoxylin & eosin (H&E) or Massons trichrome stain using standard procedures. Immuno-histochemical staining of paraffin or frozen sections was performed using the following antibodies: von Willebrand factor (vWF) (Dako North America Inc., Carpinteria, CA), keratin-14 (K14) (Covance Inc., New Jersey), Collagen IV (Acris Antibodies, San Diego, CA) after heat-induced epitope retrieval when necessary. Fluorescence detection and counterstaining were performed with Alexa Fluor 488 or 568 secondary antibody (1:200, Life Technologies, Grand Island, NY).

### Microscopy

Mosaic images were collected using a Zeiss Axiovert 200 inverted fluorescence microscope supported by an AxioCam digital camera, a motorized stage, and guided by Axiovision software (Zeiss, Thornwood, NY). Each mosaic image was generated by combining a minimum of ~100 images. Images were created by merging serial scans of thick tissue sections (20 μm) [14–17].

### Wound Planimetry

Wounds were photographed using a Canon S110 digital camera, with electro-focus and a 5.2–26.0 mm lens. A ruler was captured in the photographs near the wound border for scale adjustments. The photos were uploaded to the ImageJ software to calculate the wound area. The ruler photographed on the image was used to calibrate the scale then freehand tracing around the wound was performed [14–17].

### *Ex vivo* Tensile Strength and Dynamic Mechanical Properties Measurement

To measure the strength of the healed tissue and skin; biopsies (2” × 0.08”) were collected from the dorsum of the pig on day 14 and day 42 post-burn. The wound sites were positioned centrally within the skin biopsies as described previously [18]. Tissue specimens were mounted into the grips in to a mechanical tester (TestResources, Shakopee, MN, USA). MTestWr Version 1.3.6 software (TestResources, Shakopee, MN) was used. All skin samples were tested to failure at a strain rate of 1.3mm/s (0.05 in/s) (n = 4 per time point). To examine the dynamic mechanical properties of the healing wounds at days 14 and 42 post burning cyclic tensile tests were performed. Specimens were made as above and cyclically strained at 0.05 in/sec following a sine wave with a maximum strain of 10% and a minimum strain of 0% for a total of 20 cycles (n = 3 per time point). The resultant hysteresis in the load vs. position plots were quantified and average energy dissipation reported which is inversely related to skin elasticity. The total area within the curve between cycle 1 and cycle 20 (hysteresis) was calculated by the Trapezoid Rule using MATLAB R2014b (MathWorks, Natick, MA, USA). Average energy dissipation (mJ/mm^2^) for each time point was presented in comparison to normal pig skin from the same animal.

### Ultrasound Data Acquisition and Analysis

#### B-mode Image Acquisition and Processing

Video clips of the axial view of the wound area were recorded using a linear array probe with a frequency range of 3–18 MHz (Noblus ultrasound imaging system, Hitachi-Aloka Medical Corporation, Chiyoda-ku, Japan). A 6.5 mHz frequency was used for all the measurements described in this work. Initial adjustments were made to optimize the instrument before imaging was performed. Images/videos were recorded while gently sliding the probe head across the wound surface. Central time frames were chosen from the recorded video to measure the wound depth using the ‘measure’ feature in the software. Normal skin images were used to measure baseline skin thickness. The skin-adipose border being the brightest was used as an anatomical landmark to measure the wound depth and skin thickness. Wound depth was measured by choosing a frame that represents the wound center and applying the in-built software to obtain values depicted in the graph.

#### Color Doppler Flow Imaging

The tissue Doppler color flow imaging (CFI) feature of the machine was used to acquire video clips with color coded images representing blood flow. This technique is capable of detecting arteries and measuring maximum flow rate in the skin and wound regions on B-mode. Application of a pseudo-color to the images allows the identification of the direction of flow—forward (red) and backward (blue). Noise caused by motion was minimized by reducing pressure applied while scanning and adjusting the gain. Proper orientation and rotation of the probe enabled detection of cross-sectional and longitudinal blood vessels across the wound tissue. The identification of vessels by pulse wave Doppler velocity measurement was based on the sensitivity and specificity of the probes to detect the most prominent signal in the wound vicinity. We used the following parameters to maintain consistency of the results obtained: 1) Depth as basis for vessel identification: Measurements were made upto a 12 mm depth from the skin surface and maintained for all time points. 2) Measurement taken: Specific side of the wound was chosen for the detection of feeder vessels and kept consistent for every time point measured. 3) Size of the vessel detected: Using the length measurement tool the size of the vessels detected was measured and size matched vessels were used for the flow measurement.

#### Pulse Wave Doppler Velocity Measurement

The pulse wave color Doppler feature of the machine generated the blood flow velocity from the color flow images. Real-time velocity profiles were recorded and used to measure systole (profile peaks) and diastole (profile troughs) values. Vessel diameters were measured to identify similar blood vessels in the desired area for all time points to maintain consistency. Velocity was calculated from three peaks and troughs and the mean and standard deviation was calculated. Using the modified Bernoulli equation [19],

$$\text{P}=\frac{1}{2}\rho \left(V_{s}^{2}-V_{d}^{2}\right)$$

Where *ρ* is the density of blood, V_s_ is the velocity at systole and V_d_ is the velocity at diastole, the pulse pressure of the feeder artery was calculated.

#### Elastography and Tissue Stiffness Mapping

Tissue Doppler elastography imaging (TDI) allowed the non-invasive mapping of tissue stiffness [20]. Elastography images show a color map representing the range of tissue elasticity within the strain curve of ± 0.5 limited by the ultrasound software. Blue represents tissues with least compressibility and red represents tissues with most compressibility indicating hard to soft tissue, respectively. TDI was used to obtain maps of normal, healing and scar tissues over time. Elastography is a qualitative measurement of hardness of tissue and is assessed visually by color coding. Numeric quantification was beyond the scope of this software.

#### Scar Thickness Measurement

Tissue that was visualized as dark blue by TDI (indicating hardness) was determined to be scar tissue. Scar thickness was measured using the depth measurement feature of the software on the color elastography [21].

### Laser Speckle Perfusion Imaging and Processing

Color coded perfusion maps were acquired at all time points and average perfusion was calculated using PimSoft v1.4 software (Perimed Inc., Sweden). The wound edge and wound bed tissue regions were chosen as region of interests (ROI). From the real-time graphs obtained, time-of-interest (TOI) was chosen to include lower peak regions and to exclude motion related artifacts. Mean and standard deviation of perfusion data were obtained from the selected TOI perfusion data.

### Statistics

Data are reported as mean ± standard deviation of three wounds. Difference between means was tested using Students t-test or analysis of variance as appropriate.

## Results

### Digital image planimetry and ultrasound B-mode imaging helps visualize the progress of wound healing on a real-time basis

**Fig 1:**
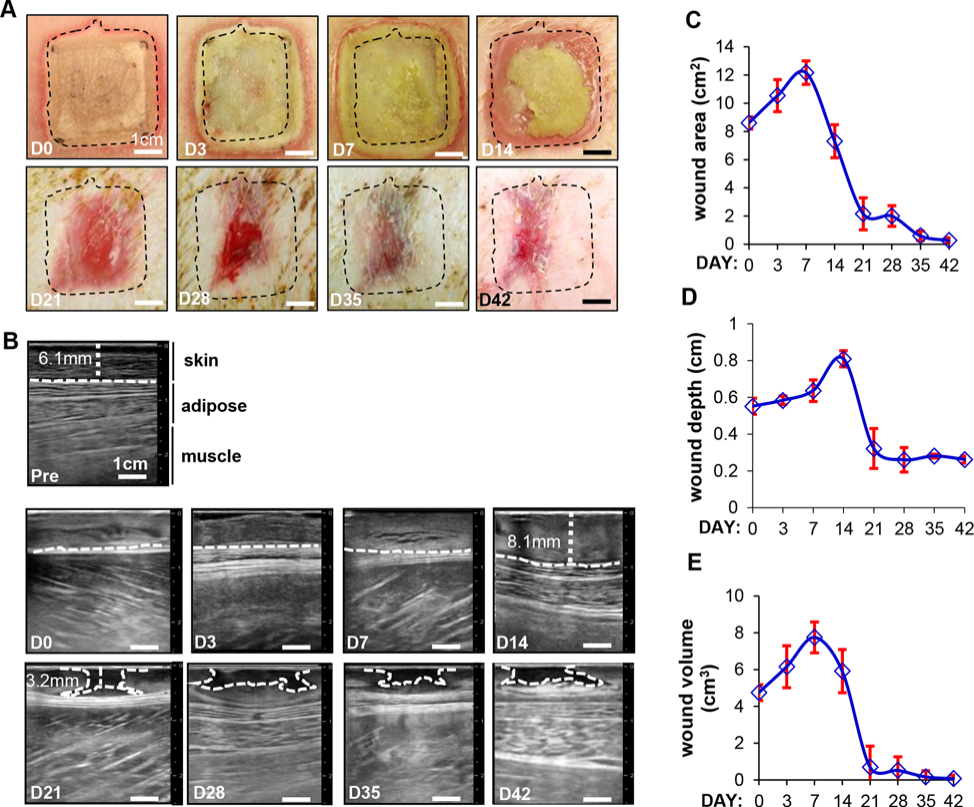
Digital image planimetry and ultrasound B-mode imaging helps visualize the progress of wound healing on a real-time basis. (A) Digital images showing a time course of wound healing starting at day 0 (immediately post-burn) and ending at day 42. Hashed line box of size 1”×1” was drawn on d0 wounds to show the actual wound size. (B) Ultrasound based axial B-mode images from the time course of the study are shown. Included is a baseline image from the normal skin pre-burn (pre). The hashed lines indicated in the images—pre d14, represent the distance of the subcutaneous tissue from the epidermal layer. The lines in images d21- d42 outline the cavitation area visualized by this imaging. (C) Image planimetry data was plotted over the time course of the study.Data are mean ± SD, n = 3 pigs. (Scale bar = 1cm). (D) Wound depth quantification was performed based on the B-mode images for all time points and represented graphically. Pre-burn images were used to measure baseline skin thickness. (E) Using the area and depth measurements, wound volume was calculated and represented graphically. Data presented as mean ± SD. (Scale bar = 1 cm).

Wound surface area was calculated using digital image planimetry. Digital images taken immediately following burn injury on the dorsum of the pig are shown in Fig. 1B (day 0) and S1 Fig. These wounds were imaged on days 3–42 and representative images are shown in Fig. 1B. Visual observations of the wound area show that there was an initial expansion of the wound area until day 7, followed by significant reduction between days 14 to 42 (Fig. 1B, dotted rectangles mark the area of the wound). This is shown quantitatively in Fig. 1C. The Noblus ultrasound scanner was used in tandem with a 5–18 MHz, 4 cm x 1cm linear array probe. The B-mode imaging feature was used to obtain cross-sectional images of the skin for longitudinal assessment of burn wounds in pigs for the first time (Fig. 1B and S1 Video). Baseline measurements of the skin (Fig. 1B, pre) allowed clear visualization and differentiation of the skin, adipose tissue and muscle layers. Immediately following the burn, these layers are no longer distinguishable (Fig. 1B, day 0). Images from day 3 to day 42 show a steady progress in the healing of the wound with an appreciable restoration of the different layers to that of baseline at day 42 (Fig. 1B, d3—d42). The lack of biomechanical properties of the underlying skin results in a concave appearance in B-mode starting at d14. The images obtained were then used to calculate wound depth which is quantitatively represented in Fig. 1D. The data shows that wound depth increased until day 14 (8.1mm) followed by a steady decrease as the healing progressed upto the end of the study. Interestingly, on days 21–42, there appears to be a cavitation visible in the healing skin layers (outlined with a white hashed line in these images), with a narrow top and a broader base region that would not be otherwise visualized by standard methods. This cavitation area was visualized in wounds from all pigs used in the study (representative images from n = 1 pig shown) with differences in size and shape as may be expected due to biological variations. From the measured wound depth and wound area, wound volume was calculated. The results show that there was a regression of the wound after three weeks (Fig. 1E).

### Histological characterization of wound healing

**Fig 2:**
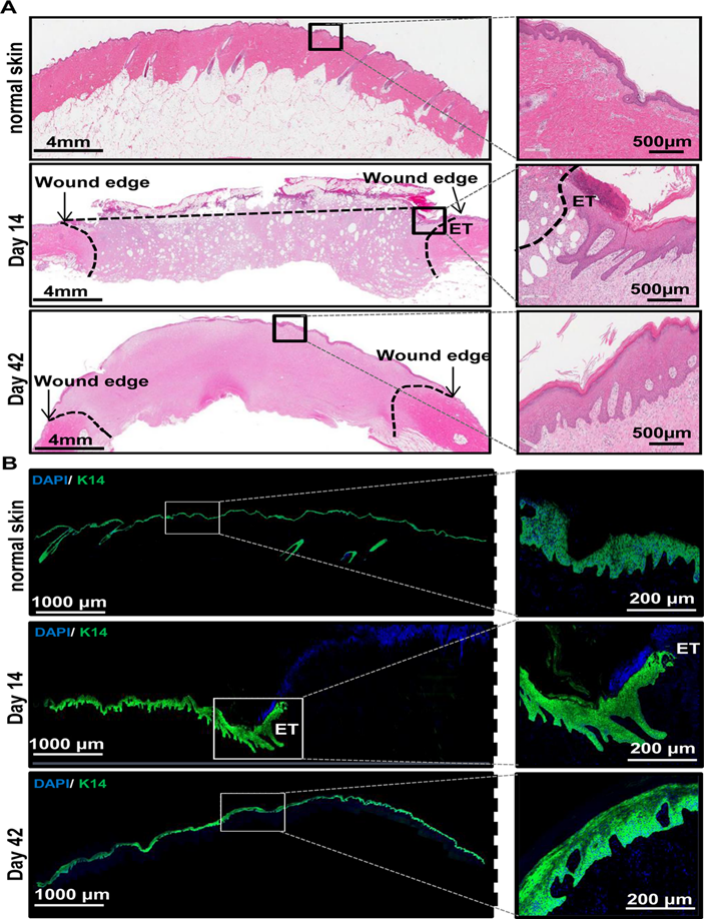
Histological characterization of wound healing. (A) Shown are representative images (left panels) from formalin-fixed paraffin-embedded biopsy tissue sections (10 μm) of normal and wounded skin (days 14 and 42) that were immunostained using hematoxylin (blue) and eosin (red). Zoomed in images (right panels) of areas in each sections are also shown for better visualization of the epithelial layer of the skin. (Scale bar = 4 mm (left panels) or 500 μm (right panels)). (B) OCT embedded frozen wound biopsies were sectioned (10 μm) and stained using anti—keratin-14 (green) and DAPI (blue). Shown are representative images (left panels) of the stained tissue sections from normal skin and wounded skin (days 14 and 42). Also shown are zoomed in images (right panels) of areas in each section for better visualization of K14 stained epithelial layer of the skin. ET = epithelial tongue. (Scale bar = 1000 μm (left panels) or 200 μm (right panels)).

Formalin-fixed paraffin sections were de-paraffinized and stained with hematoxylin and eosin (Fig. 2A). OCT embedded frozen sections were fixed and stained for a marker of epithelial cells (keratin—14) (K14; Fig. 2B). H & E and K14 (green) stained sections of normal skin and wounds from day 14 and 42 are shown in Fig. 2. On day 14 post burn, the epithelium was still in the process of being reformed and appears incomplete compared to normal skin (Fig. 2A, compare normal skin and day 14 panels). Shown in the zoomed in image is the area in the healing wound where epithelialization has started and a characteristic epithelial tongue (ET) is visible (indicated in Fig. 2A, B). This is further corroborated by the partial K14 staining in sections from day 14 wounds, where the epithelium appears to be still in the process of being formed. Also shown in these sections are DAPI stained nuclei in blue (Fig. 2B, compare normal skin and day 14 panels). Images from day 42 post burn show almost complete re-epithelialization of the wound by H&E (Fig. 2A, day 42 panels) and K14 staining (Fig. 2B, day 42 panels) indicating normal healing of the wound. However, despite re-epithelialization at 6 weeks post burn, the healing skin is still in the process of remodeling as evidenced by histological differences compared to normal skin.

### Laser speckle perfusion imaging shows dynamic changes in wound-site blood flow over time

**Fig 3:**
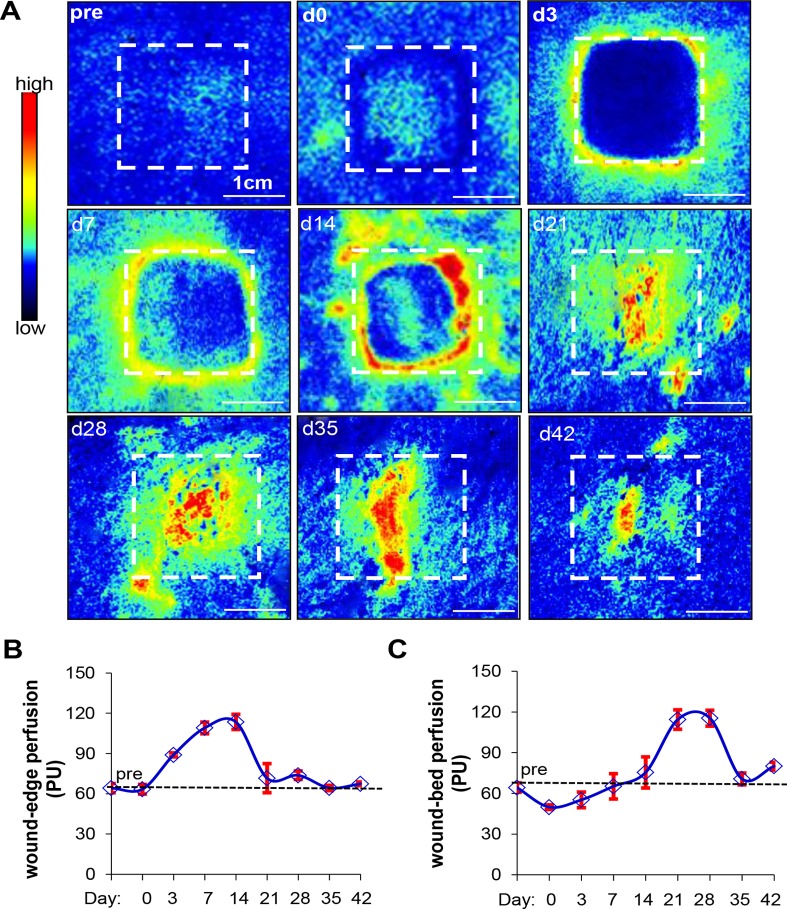
Laser speckle perfusion imaging shows dynamic changes in wound-site blood flow over time. (A) Perfusion was visualized as a two-dimensional color-coded map of blood flow (red = high; blue = low). Perfusion maps were acquired for all time points. A hashed line box representing the original wound size (1”x1”) was drawn on perfusion images to show changes in perfusion and wound size over time. (B and C) Mean perfusion at the wound edge (B) and wound bed (C) from all the time points are shown in the line graph. Data represent mean ± SD. (Scale bar = 1cm). (n = 3 pigs).

The laser speckle perfusion method was used to functionally assess vascularization in the healing wound. Measurements taken immediately before and after the burn show low baseline levels of perfusion in the wound area (indicated by the box). The perfusion maps in Fig. 3A show the temporal changes in vascularization along the wound edge (indicated by white hashed line) and wound bed through the time of study. On day 3, vasodilation of existing vessels at the periphery of the wound results in detectable perfusion that remains elevated and interestingly, appear to be confined to the edge of the wound until day 14. From d7–14, neovascularization dominates at the wound edge. Following this, concurrent with the increased perfusion in the wound bed, there is a regression of perfusion along the wound edge at day 21. Finally, by day 42, there is sharp regression of perfusion throughout the wound. This is quantitatively represented in the graphs shown in Fig. 3B and C indicating dynamic changes in the microcirculation in response to the healing process of the wound.

### Von Willebrand's factor and Collagen IV staining corroborate tissue perfusion imaging observations

**Fig 4:**
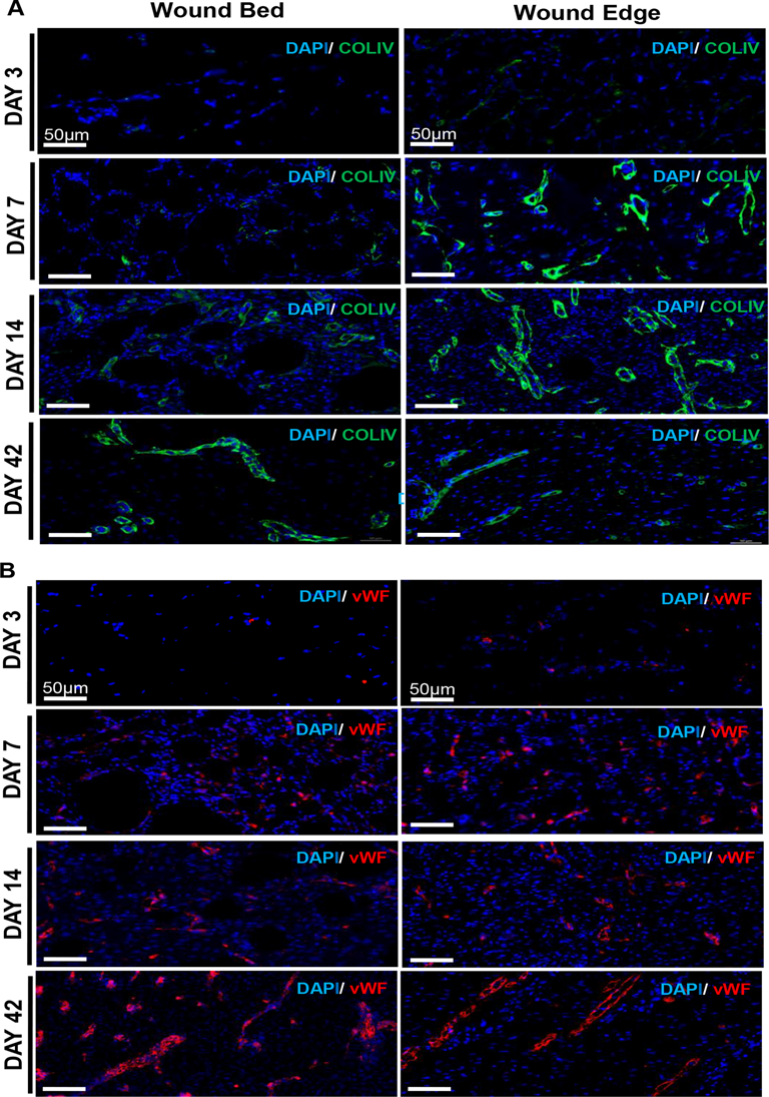
von Willebrand’s Factor and Collagen IV staining corroborate tissue perfusion imaging observations. OCT embedded frozen wound biopsies were sectioned (10 μm) and stained using anti-ColIV (green), anti-vWF (red) and DAPI (blue). Shown are representative images of the stained tissue sections from the edge and bed of the wound on days 3, 7, 14 and 42. (Scale bar = 500 μm)

Von Willebrand’s factor (vWF) is produced by endothelial cells and is a marker for vascular structures [22–24]. Collagen IV (ColIV) is deposited by endothelial cells and plays a critical role in angiogenesis [25,26]. These two markers were used in this study to visualize wound tissue vascularization on days 3, 7, 14 and 42 post burn (Fig. 4A & B). On day 3, ColIV and vWF stained structures were not visible in the wound edge or bed sections indicating that vasodilation from existing vessels near the wound edge rather than neoangiogenesis may be responsible for perfusion detected by laser speckle studies (Fig. 3). On days 7 and 14 post-burn, concurrent with the onset of angiogenesis near the wound edge, ColIV and vWF stained structures were visible in wound edge sections (Fig. 4A & B). At day 42, vWF-positive and ColIV-positive vascular structures with patent lumen were visible at both the wound bed and edge (Fig. 4A & B, day 42 panels).

### Ultrasound measurement of pulse pressure indicates enhanced blood flow *via* feeder artery supplying the edge of the wound

**Fig 5:**
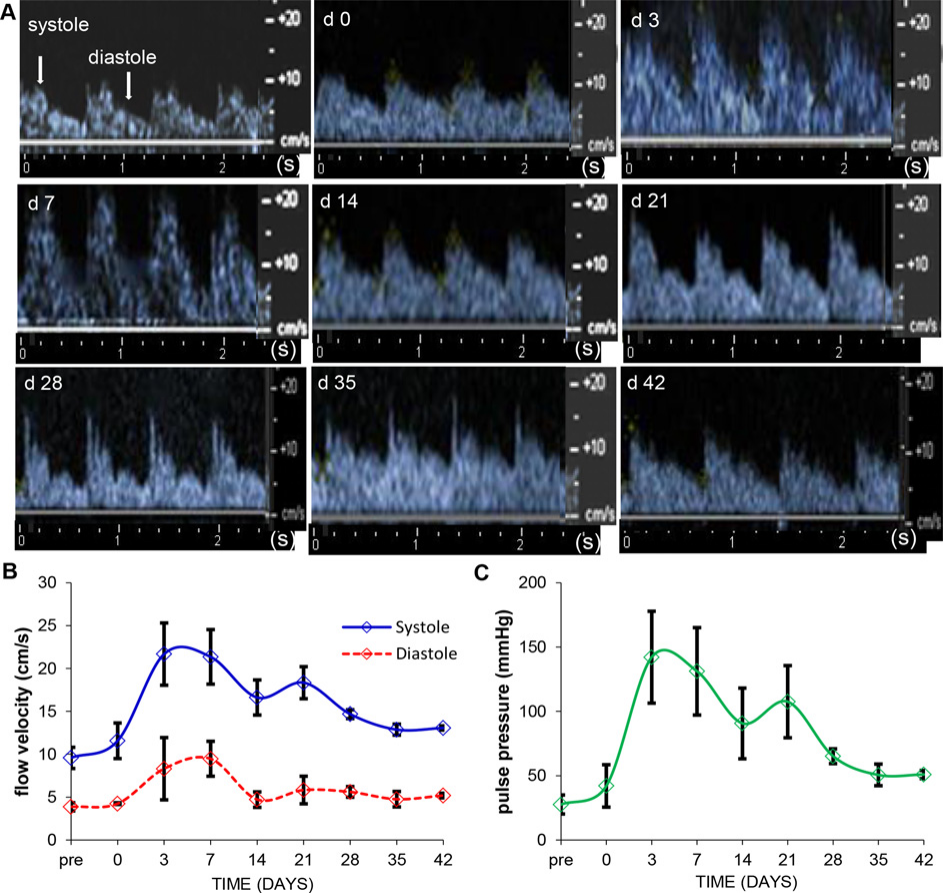
Ultrasound measurement of pulse pressure indicates enhanced blood flow *via* feeder vessels supplying the edge of the wound. (A) Velocity profile images of arterial wound-edge blood flow as measured by pulse wave Doppler flow are shown. Profiles of diastole (peaks) and systole (troughs) as indicated by arrows. (B) Flow velocities measured using the caliper feature of the software (cm/s) were plotted over time in the graph. (n = 3 pigs) (C) Mean arterial pulse pressure (mm Hg) of vessels feeding the wound area were calculated using Bernoulli’s modified hemodynamics equation and represented graphically (n = 3 pigs). Data represent mean ± SD.

To further investigate wound-edge angiogenesis, color Doppler flow imaging (CFI) was performed using harmonics ultrasound technique at the wound edge. Blood vessels were identified using CFI mode. The systole and diastole velocity profiles of feeder vessels to the wound site were obtained using pulse wave Doppler technique (representative images in Fig. 5A and S2 Video). Quantitation of the systolic and diastolic flow velocity are represented in the graph in Fig. 5B and showed that blood flow in the vessels at wound edge increased after day 3 post-burn and returned to normal values by day 42. Bernoulli’s modified hemodynamics equation (see materials and methods) was used to calculate arterial pulse pressure [19]. Interestingly, we were led to the maiden observation that the measured pulse pressure has a biphasic mode with a dramatic increase on day 3 and a shorter spike on day 21. The pressure returns to near baseline levels by day 28, indicating vascular homeostasis at this stage in the healing process (Fig. 5C).

### Tissue elastography enabled visualization of the existing and nascent tissue color-coded for their biomechanical properties

**Fig 6:**
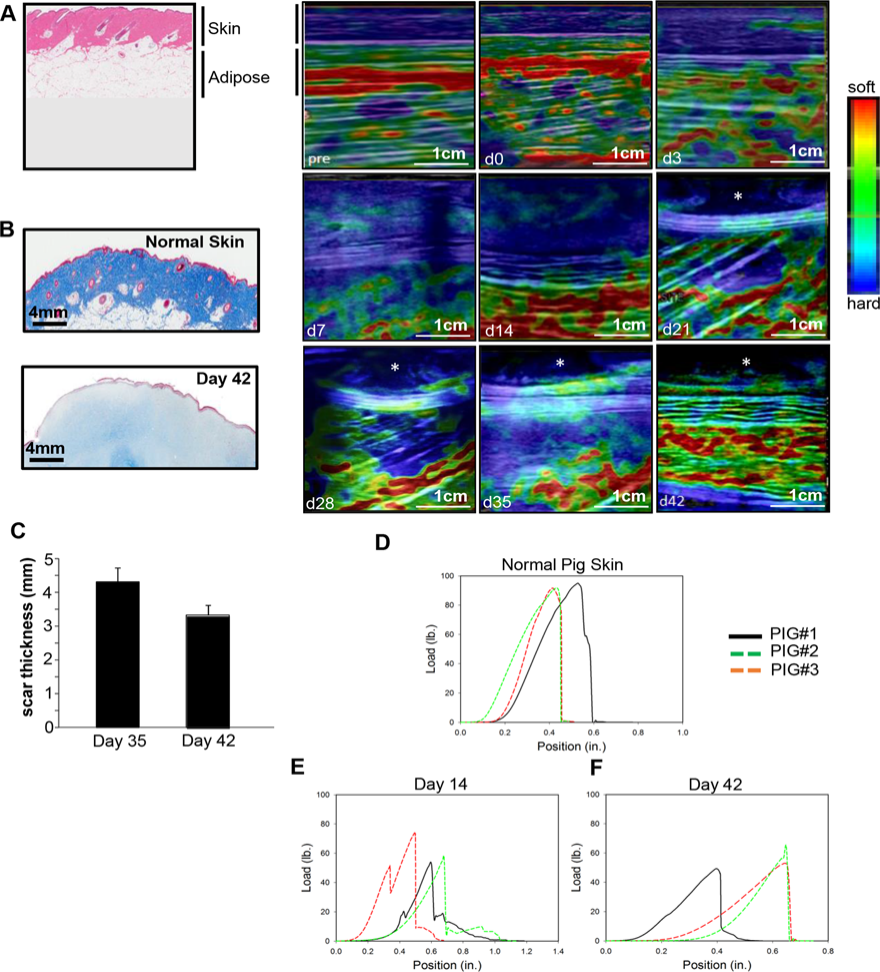
Tissue elastography enabled visualization of the existing and nascent tissue color-coded for their biomechanical properties. Skin hardness and elasticity of the wound was mapped over time as the wound heals. (A) Shown are color maps of the elasticity of the wound tissue over time. Asterisks (d21—d42 images) mark the presence of cavitation area. (n = 3 pigs). (B) Shown are representative images of formalin-fixed paraffin-embedded biopsy tissue sections (5 μm) of normal and wounded skin (Day 42) that were stained using Massons trichrome method. Staining results in blue-black nuclei, blue collagen and light red/pink cytoplasm. Epidermal cells appear red. Scale bar = 4mm. (C) Quantification of the thickness of the scar from day 35 and 42 are shown graphically. (D-F) The strength of the healing burn wounds were assessed using a TestResources mechanical tester. All skin samples were tested to failure at a strain rate of 0.05 in/sec. Load versus position for each group (normal skin, d14 and d42) is plotted; (n = 3 pigs); (Scale bar = 1cm).

Tissue stiffness *in vivo* was mapped using the TDI feature. Color maps of these measurements illustrated in Fig. 6 show variations in tissue stiffness from hard (blue) to soft (red). TDI measurements taken immediately before and after the burn (pre and day 0 respectively) indicate a very stiff outer layer (skin) and softer inner layers (adipose) (Fig. 6A). Starting at day 3 post-burn and up to day 14, corresponding with the increased wound depth, there is a decrease in the stiffness of the skin as indicated by the lighter blue and green areas in the top layers of the healing wound. From days 21 to 42, there was a progressive increase in the stiffness of the skin layers (dark blue areas). Interestingly, the cavitation visualized using B-mode imaging (Fig. 1B) was more apparent in the elastography images (marked with asterisks). Histological analysis of d42 wound indicated a lower abundance of mature collagen fibers compared to normal skin (Fig. 6B). An invasive measurement involving energy dissipation, which is inversely related to elasticity, was calculated during cyclic tensile testing. This showed a 4-fold increase in energy dissipation (decreased elasticity) from day 14 to day 42 post burn wound (average at d14 = 0.12 mJ/mm^2^, average at d42 = 0.48mJ/mm^2^). Energy dissipation from normal pig skin was significantly higher with an average of 0.71 mJ/mm^2^ (data not shown). Additionally, using elastography imaging, scar thickness was visualized and measured starting at day 35 (Fig. 6C). Measurements of the thickness of the scar using inbuilt software showed a decrease in size from days 35 and 42. Finally, tensile testing of the wound on days 14 and 42 was performed and showed that there was a decrease in failure properties of the healing skin compared to normal skin (Fig. 6D-F). Failure results also showed that the strength of the wound showed no significant improvement between 14 and 42 days of healing with an average maximum load at failure 61.3 ± 10.9 lbs. and 54.14 ± 5.5 lbs., respectively (Fig. 6E,F). While no significant differences in wound strength or stiffness in tension were observed between days 14 and 42 post burn, a larger number of delamination events, evidenced by the shoulders in the load-position curve, were present at day 14 likely indicative of an immature basement membrane formation between the epidermis and dermis (Fig. 6E).

## Discussion

This work establishes that high resolution harmonics ultrasound imaging in tandem with laser speckle flowmetry imaging is a powerful approach to longitudinally study functional wound healing non-invasively. Harmonics ultrasound imaging afforded the ability to perform high resolution, live imaging of skin and skin-associated tissues with the added advantage of studying gated feeder vessels supplying the wound site. The technologies implemented in this work allowed the monitoring and assessment of a cutaneous wound in all three dimensions affording the opportunity to scale depth. Ultrasound based B-mode imaging along with elastography measured the changes in anatomical structure as discriminated by their acoustic properties. Previous attempts to estimate wound depth have been riddled with technical challenges primarily because of the approach utilized [27,28]. The design of linear array probe taken together with the versatility of the post-processing software used in the current study provided reliable quantitation and visualization of the heterogenous wound tissue as a function of healing time. In the current experimental model of burn wounding, it took 6 weeks for the injured skin to approach baseline anatomical profile with clear presence of scar tissue. This observation is consistent with the established notion that scar tissue may take several months or years to remodel [29–33]. Indeed, a tight match between the ultrasound and histological images validates the suitability of the 6.5 MHz probe in weighing out resolution versus field of view. This probe setting should be adjusted such that higher frequency may be preferred for smaller wounds. Images shown in the current work have been subjected to the handicap of low frequency probe to make sure that the findings are relevant to clinically presented wound sizes.

In the skin, pathologies are commonly associated with changes in tissue stiffness. The ability to map elastic properties of the repairing skin enabled visualization of soft tissue growth within a week of injury. Commonly used for breast examination [34,35], this work provides first report on strain imaging and shear wave elastography of the healing wound. Measurement of the hysteresis of the load-position curve provided energy dissipation values and an invasive measurement of the elasticity of the tissue which indicated that the skin in the healing wound is more pliable or elastic at d14 compared to d42. However, even at d42, the elastic properties of the skin have not yet returned to normal skin values. Caution must be taken when comparing mechanical data from these different testing modalities, however, as they probe different volumes of the tissue and probe the tissue using different forces (tension versus compression etc.). Elastography also enabled the visualization and quantification of post-closure scar tissue. The results are in tight agreement with histological findings. During the first two weeks after injury, it is visible that the subcutaneous tissue is depressed by an expanding presence of granulation tissue which serves as the site of inflammation. One week thereafter, the granulation tissue shrinks making room for the subcutaneous tissue to return to its prior position. At the same time, appearance of the leading edge from both sides is evident. Upon closure in the 6^th^ week after injury, the skin and subcutaneous adipose tissues re-position in a way that closely resembles but does not exactly match the baseline skin image. The cavitation noted on the closed wound represents the scar tissue as validated histologically. It is possible that this cavitation area in the wound might represent a region where extracellular matrix remodeling is still occurring, therefore having lower collagen density. This is corroborated by the lower abundance of mature collagen fibers in d42 wounds compared to normal skin. This could compromise the biomechanical properties or elasticity of the wound, making it brittle and compromising its load bearing capacity. In the context of findings related to post-closure tensile strength, the implications of this observation are that the cavitation area may be a vulnerable point in the wound and likely to be more prone to wound recurrence.

The ultrasound transducer used in the current study is capable of Doppler color flow imaging. While this technology platform is commonly used for diagnostic echocardiography [36,37], this work provides first evidence on functional blood flow parameters in gated peripheral feeder artery supplying the wound site. In isolation, angiogenic factors or endothelial cell proliferation is not sufficient to induce angiogenesis. It is well documented that hemodynamic factors play a key role in driving inducible angiogenesis. Importantly, these biomechanical forces have to work synergistically with chemical factors in order to drive the proper establishment of vascular supply. A combination of biomechanical stimulation (*e*.*g*. shear stress, circumferential stretch and mechanotransduction) [38–44] and chemical stimulation (*e*.*g*. angiogenic proteins and growth factors) [45–50] orchestrate various aspects of neo-vascularization including the proliferation of cells (endothelial, smooth muscle and fibroblasts), regulation of permeability, stabilization of vessels and the production of the extracellular matrix (ECM). Modulation of simply one or the other of these regulatory arms may be insufficient to trigger functional angiogenesis to the full extent possible. This is evidenced from reports of gene therapies targeting the vascular endothelial growth factor (VEGF) that have failed in clinical trials [51,52] possibly because they target only one aspect of the above mentioned combinatorial regulatory process. Because of technological limitations currently there is no functional evidence in the literature as to how wound angiogenesis is related to changes in blood flow velocity of the primary feeder artery that supplies the wound site. While pulse velocity is commonly used to assess arterial wall stiffness, it is also a key determinant of local hemodynamic performance. Higher pulse velocity can only be generated by healthy arteries and will propel blood flow within the given vessel resulting in higher sheer stress which in turn is likely to drive wound angiogenesis. As expected, pulse velocity was recorded as being low, comparable to that of homeostatic baseline skin, immediately post-injury. Hypothetically one of the earliest drivers of wound angiogenesis is a sharp elevation of pulse velocity in the primary feeder artery that supplies the wound site. This remarkable change is noted on day 3 at the inflammatory phase as blood borne immune cells accumulate at the wound site. The mechanisms underlying this escalation remain unknown. During the course of the next two weeks of the healing process there appears to be a correction of pulse velocity wherein the velocity is still 5-fold of the baseline but has declined by about a third of where it was during the peak on day 3. This observation leads to the speculation that the noted rise in pulse velocity after wounding is not completely dependent on cells abundant during the inflammatory phase. As evident histologically with this wound model, the inflammatory phase has been largely resolved by the end of the second week. Of outstanding interest is the observation that as pulse velocity declines from day 3 to day 14, the system engages in a second boost of pulse velocity resulting in a bimodal peak as reported. This tight dual control of the arterial pulse velocity points towards an extraordinary significance of arterial hemodynamics in wound angiogenesis.

Laser speckle imaging has previously been used for the assessment of spatio-temporal hemodynamic changes during excisional wound healing [53,54]. We were able to visualize perfusion changes in the entire wound area as healing progressed and these were validated with histological analyses. Perfusion is visibly increased along the wound edge during early stages of wound healing, co-inciding with the inflammatory phase of wound healing, which then proceeds to the wound bed as healing progresses. ColIV has been implicated as an early marker of neoangiogenesis in recent studies on rat gastric walls [55]. ColIV is detected earlier during wound healing than vWF. As tissue remodeling occurs (d42), perfusion in the wound edge regresses and returns to baseline levels comparable to that of the surrounding normal skin [56,57]. Remarkably, it appears that it takes over a month for angiogenesis induced at wound edge (d3) to resolve (d42) and approach baseline levels. Although similar spatial changes in perfusion have been previously noted in short term studies on excisional wound healing [53,54], this study is the first to longitudinally follow the spatio-temporal changes in blood perfusion over a period of six weeks.

In conclusion, we demonstrate that harmonic ultrasound in tandem with laser speckle technologies represent a powerful approach for the non-invasive longitudinal assessment of the cutaneous wound healing process with higher resolution and accuracy. This approach has the power to comprehensively query wound depth and tunneling issues that are of critical clinical significance. The utility of these powerful tools in visualizing and characterizing wound healing multi-dimensionally, without the need for invasive measures is an incomparable advantage for both clinical as well as research applications. Visualization of the longitudinal cutaneous wound healing process through the lens of the above-mentioned technologies provide the opportunity to appreciate mechanistic underpinnings that are otherwise not evident. Changes in the pulse velocity pattern in the primary feeder artery supplying the wound site, is one such example. In summary, this work establishes that high-resolution harmonics ultrasound imaging is a powerful approach for the non-invasive characterization of cutaneous wound healing.

## Supporting Information

S1 Fig Digital images of 1”x1” burn wounds applied to the dorsum of pig. (TIF)

S1 Video Ultrasound B-mode imaging showing normal and wound areas on day 14 post-burn. (WMV)

S2 Video Color Doppler flow imaging of a feeder vessel showing measurement of systolic and diastolic pressures. (WMV)

## References

[1] Brigham PA, McLoughlin E. Burn incidence and medical care use in the United States: estimates, trends, and data sources. J Burn Care Rehabil. 1996; 17: 95–107. 8675512 10.1097/00004630-199603000-00003

[2] Sen CK, Gordillo GM, Roy S, Kirsner R, Lambert L, Hunt TK, et al Human skin wounds: a major and snowballing threat to public health and the economy. Wound Repair Regen. 2009; 17: 763–771. 10.1111/j.1524-475X.2009.00543.x 19903300 PMC2810192

[3] Monstrey S, Hoeksema H, Verbelen J, Pirayesh A, Blondeel P. Assessment of burn depth and burn wound healing potential. Burns. 2008; 34: 761–769. 10.1016/j.burns.2008.01.009 18511202

[4] Davis SC, Pogue BW, Springett R, Leussler C, Mazurkewitz P, Tuttle SP, et al Magnetic resonance-coupled fluorescence tomography scanner for molecular imaging of tissue. Rev Sci Instrum. 2008; 79: 064302 10.1063/1.2919131 18601421 PMC2678791

[5] D'Astous FT, Foster FS. Frequency dependence of ultrasound attenuation and backscatter in breast tissue. Ultrasound Med Biol. 1986; 12: 795–808. 3541334 10.1016/0301-5629(86)90077-3

[6] Manduca A, Oliphant TE, Dresner MA, Mahowald JL, Kruse SA, Amromin E, et al Magnetic resonance elastography: non-invasive mapping of tissue elasticity. Med Image Anal. 2001; 5: 237–254. 11731304 10.1016/s1361-8415(00)00039-6

[7] Peeters W, Anthonissen M, Deliaert A, Van der Hulst R, Van den Kerckhove E . A comparison between laser-doppler imaging and colorimetry in the assessment of scarring: "a pilot study". Skin Res Technol. 2012; 18: 188–191. 10.1111/j.1600-0846.2011.00552.x 22092605

[8] Limbourg A, Korff T, Napp LC, Schaper W, Drexler H, Limbourg FP. Evaluation of postnatal arteriogenesis and angiogenesis in a mouse model of hind-limb ischemia. Nat Protoc. 2009; 4: 1737–1746. 10.1038/nprot.2009.185 19893509

[9] Forrester KR, Tulip J, Leonard C, Stewart C, Bray RC. A laser speckle imaging technique for measuring tissue perfusion. IEEE Trans Biomed Eng. 2004; 51: 2074–2084. 15536909 10.1109/TBME.2004.834259

[10] Martin K. Introduction to B-mode imaging In: Hoskins P, Martin K., Thrush A., editor. Diagnostic Ultrasound-Physics and Equipment. 2nd ed Cambridge, UK: Cambridge University Press; 2010.

[11] Krouskop TA, Wheeler TM, Kallel F, Garra BS, Hall T. Elastic moduli of breast and prostate tissues under compression. Ultrason Imaging. 1998; 20: 260–274. 10197347 10.1177/016173469802000403

[12] Coutts L, Bamber J, Miller N. Multi-directional in vivo tensile skin stiffness measurement for the design of a reproducible tensile strain elastography protocol. Skin Res Technol. 2013; 19: e37–44. 10.1111/j.1600-0846.2011.00604.x 22309091

[13] Gennisson JL, Deffieux T, Fink M, Tanter M. Ultrasound elastography: principles and techniques. Diagn Interv Imaging. 2013; 94: 487–495. 10.1016/j.diii.2013.01.022 23619292

[14] Elgharably H, Roy S, Khanna S, Abas M, Dasghatak P, Das A, et al A modified collagen gel enhances healing outcome in a preclinical swine model of excisional wounds. Wound Repair Regen. 2013; 21: 473–481. 10.1111/wrr.12039 23607796 PMC3685858

[15] Roy S, Biswas S, Khanna S, Gordillo G, Bergdall V, Green J, et al Characterization of a preclinical model of chronic ischemic wound. Physiol Genomics. 2009; 37: 211–224. 10.1152/physiolgenomics.90362.2008 19293328 PMC2685508

[16] Roy S, Driggs J, Elgharably H, Biswas S, Findley M, Khanna S, et al Platelet-rich fibrin matrix improves wound angiogenesis via inducing endothelial cell proliferation. Wound Repair Regen. 2011; 19: 753–766. 10.1111/j.1524-475X.2011.00740.x 22092846 PMC3623798

[17] Roy S, Elgharably H, Sinha M, Ganesh K, Chaney S, Mann E, et al Mixed-species biofilm compromises wound healing by disrupting epidermal barrier function. J Pathol. 2014; 233: 331–343. 10.1002/path.4360 24771509 PMC4380277

[18] Christoforidis JB, Wang J, Jiang A, Willard J, Pratt C, Abdel-Rasoul M, et al The effect of intravitreal bevacizumab and ranibizumab on cutaneous tensile strength during wound healing. Clin Ophthalmol. 2013; 7: 185–191. 10.2147/OPTH.S40537 23378736 PMC3559083

[19] Badeer HS. Elementary hemodynamic principles based on modified Bernoulli's equation. Physiologist. 1985; 28: 41–46. 3983247

[20] Maurice RL, Daronat M, Ohayon J, Stoyanova E, Foster FS, Cloutier G. Non-invasive high-frequency vascular ultrasound elastography. Phys Med Biol. 2005; 50: 1611–1628. 15798347 10.1088/0031-9155/50/7/020

[21] Lau JC, Li-Tsang CW, Zheng YP. Application of tissue ultrasound palpation system (TUPS) in objective scar evaluation. Burns. 2005; 31: 445–452. 15896506 10.1016/j.burns.2004.07.016

[22] Jaffe EA, Hoyer LW, Nachman RL. Synthesis of von Willebrand factor by cultured human endothelial cells. Proc Natl Acad Sci U S A. 1974; 71: 1906–1909. 4209883 10.1073/pnas.71.5.1906 PMC388351

[23] Yamamoto K, de Waard V, Fearns C, Loskutoff DJ. Tissue distribution and regulation of murine von Willebrand factor gene expression in vivo. Blood. 1998; 92: 2791–2801. 9763564

[24] Muller AM, Skrzynski C, Skipka G, Muller KM. Expression of von Willebrand factor by human pulmonary endothelial cells in vivo. Respiration. 2002; 69: 526–533. 12457006 10.1159/000066471

[25] Madri JA. Extracellular matrix modulation of vascular cell behaviour. Transpl Immunol. 1997; 5: 179–183. 9402683 10.1016/s0966-3274(97)80035-4

[26] Maragoudakis ME, Missirlis E, Karakiulakis GD, Sarmonica M, Bastakis M, Tsopanoglou N. Basement membrane biosynthesis as a target for developing inhibitors of angiogenesis with anti-tumor properties. Kidney Int. 1993; 43: 147–150. 7679456 10.1038/ki.1993.24

[27] Davis KE, Constantine FC, Macaslan EC, Bills JD, Noble DL, Lavery LA. Validation of a laser-assisted wound measurement device for measuring wound volume. J Diabetes Sci Technol. 2013; 7: 1161–1166. 24124941 10.1177/193229681300700508 PMC3876358

[28] Little C, McDonald J, Jenkins MG, McCarron P. An overview of techniques used to measure wound area and volume. J Wound Care. 2009; 18: 250–253. 19661849 10.12968/jowc.2009.18.6.42804

[29] Witte MB, Barbul A. General principles of wound healing. Surg Clin North Am. 1997; 77: 509–528. 9194878 10.1016/s0039-6109(05)70566-1

[30] Hunt TK, Hopf H, Hussain Z. Physiology of wound healing. Adv Skin Wound Care. 2000; 13: 6–11. 11074996

[31] Velnar T, Bailey T, Smrkolj V. The wound healing process: an overview of the cellular and molecular mechanisms. J Int Med Res. 2009; 37: 1528–1542. 19930861 10.1177/147323000903700531

[32] Broughton G 2nd, Janis JE, Attinger CE. The basic science of wound healing. Plast Reconstr Surg. 2006; 117: 12S–34S. 16799372 10.1097/01.prs.0000225430.42531.c2

[33] Ramasastry SS. Acute wounds. Clin Plast Surg. 2005; 32: 195–208. 15814117 10.1016/j.cps.2004.12.001

[34] Berg WA, Cosgrove DO, Dore CJ, Schafer FK, Svensson WE, Hooley RJ, et al Shear-wave elastography improves the specificity of breast US: the BE1 multinational study of 939 masses. Radiology. 2012; 262: 435–449. 10.1148/radiol.11110640 22282182

[35] Cosgrove DO, Berg WA, Dore CJ, Skyba DM, Henry JP, Gay J, et al Shear wave elastography for breast masses is highly reproducible. Eur Radiol. 2012; 22: 1023–1032. 10.1007/s00330-011-2340-y 22210408 PMC3321140

[36] Ommen SR, Nishimura RA, Appleton CP, Miller FA, Oh JK, Redfield MM, et al Clinical utility of Doppler echocardiography and tissue Doppler imaging in the estimation of left ventricular filling pressures: A comparative simultaneous Doppler-catheterization study. Circulation. 2000; 102: 1788–1794. 11023933 10.1161/01.cir.102.15.1788

[37] Ommen SR, Nishimura RA, Hurrell DG, Klarich KW. Assessment of right atrial pressure with 2-dimensional and Doppler echocardiography: a simultaneous catheterization and echocardiographic study. Mayo Clin Proc. 2000; 75: 24–29. 10630753 10.4065/75.1.24

[38] Lehoux S, Castier Y, Tedgui A. Molecular mechanisms of the vascular responses to haemodynamic forces. J Intern Med. 2006; 259: 381–392. 16594906 10.1111/j.1365-2796.2006.01624.x

[39] Hoefer IE, den Adel B, Daemen MJ. Biomechanical factors as triggers of vascular growth. Cardiovasc Res. 2013; 99: 276–283. 10.1093/cvr/cvt089 23580605

[40] Boerckel JD, Uhrig BA, Willett NJ, Huebsch N, Guldberg RE. Mechanical regulation of vascular growth and tissue regeneration in vivo. Proc Natl Acad Sci U S A. 2011; 108: E674–680. 10.1073/pnas.1107019108 21876139 PMC3174614

[41] Mulvany MJ, Aalkjaer C. Structure and function of small arteries. Physiol Rev. 1990; 70: 921–961. 2217559 10.1152/physrev.1990.70.4.921

[42] Pourageaud F, De Mey JG. Structural properties of rat mesenteric small arteries after 4-wk exposure to elevated or reduced blood flow. Am J Physiol. 1997; 273: H1699–1706. 9362233 10.1152/ajpheart.1997.273.4.H1699

[43] Ingber DE, Folkman J. How does extracellular matrix control capillary morphogenesis? Cell. 1989; 58: 803–805. 2673531 10.1016/0092-8674(89)90928-8

[44] Ingber DE, Folkman J. Mechanochemical switching between growth and differentiation during fibroblast growth factor-stimulated angiogenesis in vitro: role of extracellular matrix. J Cell Biol. 1989; 109: 317–330. 2473081 10.1083/jcb.109.1.317 PMC2115480

[45] Resnick N, Gimbrone MA Jr. Hemodynamic forces are complex regulators of endothelial gene expression. FASEB J. 1995; 9: 874–882. 7615157 10.1096/fasebj.9.10.7615157

[46] Rubanyi GM, Freay AD, Kauser K, Johns A, Harder DR. Mechanoreception by the endothelium: mediators and mechanisms of pressure- and flow-induced vascular responses. Blood Vessels. 1990; 27: 246–257. 2242445 10.1159/000158816

[47] Carmeliet P. Angiogenesis in life, disease and medicine. Nature. 2005; 438: 932–936. 16355210 10.1038/nature04478

[48] Battegay EJ. Angiogenesis: mechanistic insights, neovascular diseases, and therapeutic prospects. J Mol Med (Berl). 1995; 73: 333–346. 8520966 10.1007/BF00192885

[49] Schott RJ, Morrow LA. Growth factors and angiogenesis. Cardiovasc Res. 1993; 27: 1155–1161. 7504584 10.1093/cvr/27.7.1155

[50] Adams RH, Alitalo K. Molecular regulation of angiogenesis and lymphangiogenesis. Nat Rev Mol Cell Biol 2007; 8: 464–478. 17522591 10.1038/nrm2183

[51] Stewart DJ, Kutryk MJ, Fitchett D, Freeman M, Camack N, Su Y, et al VEGF gene therapy fails to improve perfusion of ischemic myocardium in patients with advanced coronary disease: results of the NORTHERN trial. Mol Ther. 2009; 17: 1109–1115. 10.1038/mt.2009.70 19352324 PMC2835194

[52] Gupta R, Tongers J, Losordo DW. Human studies of angiogenic gene therapy. Circ Res. 2009; 105: 724–736. 10.1161/CIRCRESAHA.109.200386 19815827 PMC2770893

[53] Rege A, Thakor NV, Rhie K, Pathak AP. In vivo laser speckle imaging reveals microvascular remodeling and hemodynamic changes during wound healing angiogenesis. Angiogenesis. 2012; 15: 87–98. 10.1007/s10456-011-9245-x 22198198 PMC4380186

[54] Stewart CJ, Gallant-Behm CL, Forrester K, Tulip J, Hart DA, Bray RC. Kinetics of blood flow during healing of excisional full-thickness skin wounds in pigs as monitored by laser speckle perfusion imaging. Skin Res Technol. 2006; 12: 247–253. 17026655 10.1111/j.0909-752X.2006.00157.x

[55] Fonseca J, Martins-dos-Santos J, Oliveira P, Laranjeira N, Aguas A, Castelo-Branco N. Noise-induced gastric lesions: a light and electron microscopy study of the rat gastric wall exposed to low frequency noise. Arq Gastroenterol. 2012; 49: 82–88. 22481691 10.1590/s0004-28032012000100014

[56] Gurtner GC, Werner S, Barrandon Y, Longaker MT. Wound repair and regeneration. Nature 2008; 453: 314–321. 10.1038/nature07039 18480812

[57] Swift ME, Kleinman HK, DiPietro LA. Impaired wound repair and delayed angiogenesis in aged mice. Lab Invest. 1999; 79: 1479–1487. 10616199

