# Supplementary figures and images for: High-Resolution Harmonics Ultrasound Imaging for Non-Invasive Characterization of Wound Healing in a Pre-Clinical Swine Model
Source: PLoS One. 2015 Mar 23;10(3):e0122327. doi: 10.1371/journal.pone.0122327 (PMC4370665; doi:10.1371/journal.pone.0122327)

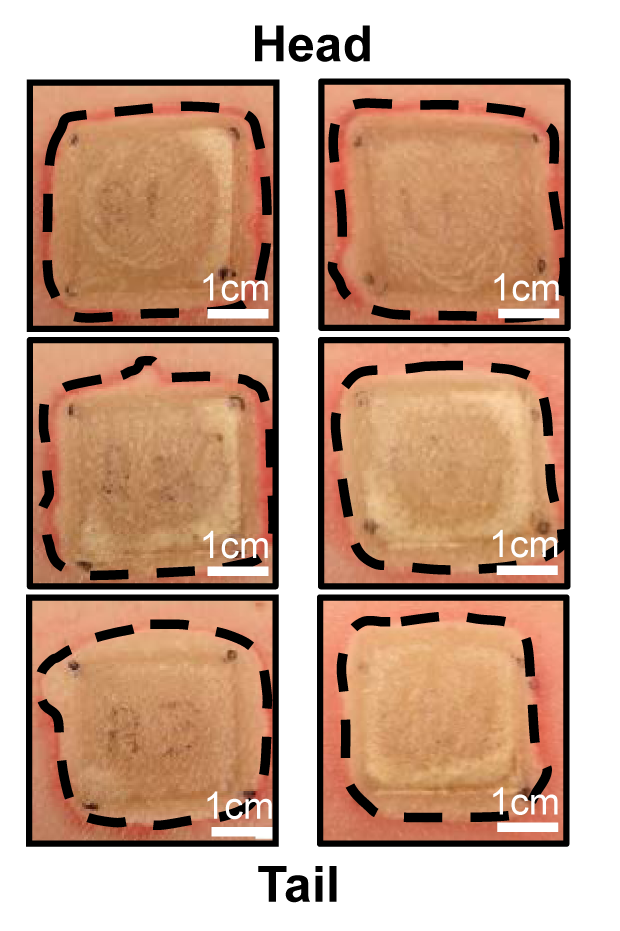

Supplement: S1 Fig — (TIF) [file pone.0122327.s001.tif]
